# Time heals. A study of thyroidectomy scars^☆^^☆☆^

**DOI:** 10.1016/j.bjorl.2025.101641

**Published:** 2025-05-29

**Authors:** Vergilius José Furtado de Araujo Filho, Vergilius José Furtado de Araujo-Neto, Leandro Luongo de Matos

**Affiliations:** Universidade de São Paulo (USP), Faculdade de Medicina (FM), Disciplina de Cirurgia de Cabeça e Pescoço, São Paulo, SP, Brazil

**Keywords:** Thyroidectomy, Thyroid gland, Cicatrix

## Abstract

**Objective:**

To evaluate the quality of the thyroidectomy scars over a long period of time.

**Methods:**

Over a period of two years the quality of the thyroidectomy scars among 283 consecutive patients was registered during their follow-up for malignancies. On a scale of 0 (excellent) to 3 (bad) thyroidectomy scars were classified according to the time after surgery.

**Results:**

After 5 years or more 95% of the scars were classified as excellent or very good; 55% of them were excellent, which indicates that the scar was virtually invisible at a distance of 1.5 meters. Only 5% of the cases had a regular or bad scar after 5-years or more, and only one patient (0.5%) had a bad scar.

**Conclusion:**

The scars remaining after thyroid surgery had an improved appearance with time, and the vast majority of patients had an excellent or very good scar after a period of five years or more.

**Level of evidence:**

Level III.

## Highlights

• 95% of the scars were classified as excellent or very good, Grade 0 or 1 in longer follow-up.
• Age, sex, hypertension, diabetes, smoking and history of hypertrophic scars did not show any significant differences.
• The cosmetic quality of scar improves with postoperative length of time.

## Introduction

Thyroidectomy is a commonly performed surgical procedure and there is a major concern among patients about the resultant scar in an exposed area of the body.

Many efforts have been made to improve the cosmetic results after thyroidectomy such as remote approaches with endoscopic or robotic techniques. The problem with these techniques is that they are not applicable to every patient or for every thyroid disease. These techniques also increase operative time, cost and probably, complication rates. Open thyroid surgery is a very safe procedure with rare but potentially devastating complications. Although experts in these remote approaches claim that the risks are almost the same, there is certainly a learning curve during which some patients will be put at a higher risk.

It has been our observation that most thyroidectomy scars just disappear with time; therefore, we question the benefits of these “new” techniques.

The purpose of this study was to evaluate the quality of the thyroidectomy scars over a long period of time.

## Methods

This study was approved by the Institutional Review Board (CAPPesq nº 0712/11).

Over a period of two years (2016–2018) we have registered the quality of the thyroidectomy scars in 283 consecutive patients during their follow-up for malignancies; most of these patients were being followed after a total thyroidectomy for papillary carcinoma (Table 1). All patients were operated on and followed by the same surgeon (VJFAF) in his private clinic.

**Table 1:** Types of operations.

	N	%
PT	10	4
TT	239	84
TT + CCND	8	3
TT + CC + LCND	26	9
TOTAL	283	100

PT, Partial Thyroidectomy; TT, Total Thyroidectomy; TT + CCND, Total Thyroidectomy and Central Compartment Neck Dissection; TT + CC + LCND, Total Thyroidectomy and Central Compartment neck dissection and Lateral Compartment Neck Dissection.

**Fig. 1:**
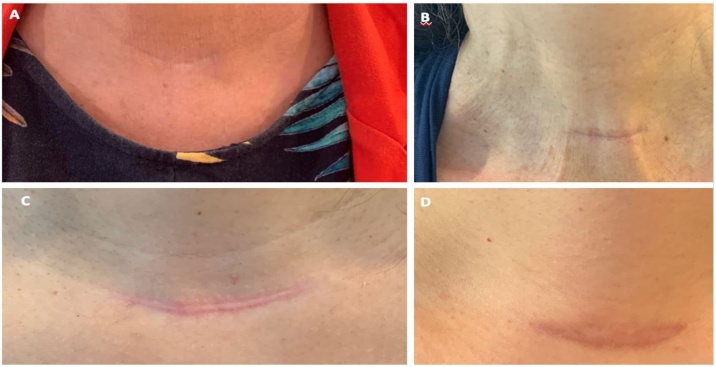
Thyroidectomy scars graded as 0 (A), 1 (B), 2 (C) and 3 (D).

Notes were taken about postoperative time and the quality of the scar. This was measured in a simple way, on a scale from 0 to 3 (Table 2 and Fig. 1). This new proposed method of scar evaluation was inspired in the cosmetic evaluation of nodular goiter.^1^ Additionally, we checked for the relationship of the scar grade to other factors, such as smoking habits, diabetes mellitus, hypertension and history of hypertrophic scars from previous surgeries.

**Table 2:** Grades of the scars.

Grade	
0	Virtually invisible at 1.5 meters of distance
1	Visible but a very good scar at 1.5 meters of distance
2	Visible regular scar at 1.5 meters of distance
3	Visible and bad (hyperchromic, hypertrophic) scar at 1.5 meters of distance

About the surgical technique for thyroidectomy, the length of the scars was from 3 to 5 cm, usually 4 cm. We continued in the plane below the platysma. Closure was made with separate subcutaneous sutures of Vicryl 4‒0 and the skin was closed with a continuous suture of Monocryl 5‒0. Drains were used only in cases for whom the lateral neck approach was used.

## Results

There were 78 men (28%) and 205 women (72%). The youngest patient was 16 years old and the oldest was 87; the mean age was 44.6 years old. The first surgery was in July 1993, and the last surgery in this series was in October 2018.

**Fig. 2:**
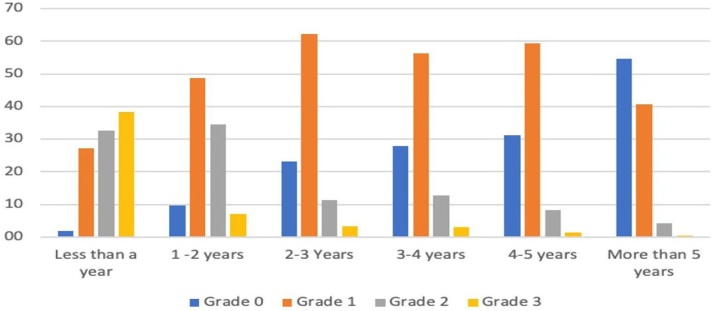
Percentage of each category of patients in each period of time.

The quality of the scar relative to postoperative time is shown in Table 3 and Fig. 2.

**Table 3:** Results of the quality of the scar by the time of observation, n (%).

	<1 Year	1‒2 Years	2‒3 Years	3‒4 Years	4‒5 Years	>5 Years
Grade 0	3 (1.9)	15 (9.6)	35 (23.2)	37 (27.8)	42 (31.1)	102 (54.5)
Grade 1	44 (27.2)	76 (48.7)	94 (62.3)	75 (56.4)	80 (59.3)	76 (40.6)
Grade 2	53 (32.7)	54 (34.6)	17 (11.3)	17 (12.8)	11 (8.1)	8 (4.3)
Grade 3	62 (38.3)	11 (7.1)	5 (3.3)	4 (3.0)	2 (1.5)	1 (0.5)

Other factors studied such as age, sex, hypertension, diabetes, smoking and history of hypertrophic scars did not show any significant differences in this series, they were considered irrelevant and are not shown here.

## Discussion

Our data from a large number of cases showed that the vast majority of the patients submitted to thyroidectomies had an excellent appearance of their scars after a period of five years or more. These results also show that time is a crucial factor for healing.

If we consider that Grade 0 is excellent, Grade 1 is very good, Grade 2 is regular and Grade 3 is bad, by the first year after the operation 71% of the scars were considered regular or bad and 38% of them bad. In contrast after 5-years or more 95% of the scars were excellent or very good, and 55% of them were excellent, which means a virtually invisible scar at 1.5 meters of distance. Only 5% of the cases had a regular or bad scar after 5-years or more and only one patient (0.5%) had a bad scar.

This is, in our opinion, the problem with the articles that have previously studied this subject. Time of follow-up was too short in most of them.

Lee et al.^2^ showed some benefits on self-body image in cases of robotic thyroidectomy compared to open thyroidectomy in 116 patients treated for papillary thyroid carcinoma. However, the period of evaluation was 9 months.

Ma et al.^3^ compared the cosmetic outcomes of 3 methods of thyroidectomy; conventional surgery, aesthetic principles access and minimally invasive approaches. In 120 female patients, better scores were in the second group, but the time of follow-up was 12.3 months.

In a study of 1141 thyroidectomies, Shin et al.^4^ found that the development of hypertrophic scars was present in 13.9% of the patients after 57.92 ± 40.7 days. They recommended early intervention by dermatologists with treatments such as laser treatment, intralesional steroid injection and topical agents. The time of follow-up was 6 months.

Perhaps because many surgeons do not follow their patients after thyroidectomy for a long time, instead leaving the follow-up for the clinicians to conduct, they only see the scars during a short postoperative time. This is only a hypothesis, but it can explain why the previous studies have been conducted with only a short period of follow up. We follow our patients for life, in addition to the follow-up conducted by the clinicians. This is the reason why there were only cases of malignancies of the thyroid in this series; the benign cases were not followed.

Arora et al.^5^ found a significant difference in the patient perception of the scar (using the VAS scale) when they compared 16 patients who underwent transaxillary robotic thyroidectomy with 16 patients who underwent conventional surgery in a UK population with a follow-up time of 4 years. The 100-rate level score in this scale is for the ideal cosmesis. The difference was 91.9 ± 11.1 vs. 78.9 ± 17.9, (p = 0.005) at 3 months and 95.5 ± 6.3 vs. 89.7 ± 8.5, (p = 0.02 at 3 years). We think that, despite the statistical significance, the difference between the groups in the later period was small, and the satisfaction of patients with their cervical incisions was very high 3 years after surgery. Our data also show that the percentage of excellent and good scars at this time of follow-up is also very high.

There are some different methods of evaluating the quality of the scars.^6^, ^7^, ^8^ They tend to be complex but are more complete when taking into account the opinion of the patients, and when the observation is made by someone other than the surgeon who performed the operation, which is an evident bias. However, those factors are also very subjective, as we are discussing aesthetics problems. We have chosen a simple method that would answer a frequent question from the patients, “what will happen with my scar?”. This can be such an important matter for anyone, especially for the group of patients who typically have to be submitted to a thyroidectomy, i.e., young women.

The search for methods that will not leave an apparent scar is in our opinion very positive. Technical innovations are welcomed and must be tried. However, the methods that we have currently imply in greater costs, and more complex approaches compared with the standard thyroidectomy, which is very safe and efficient. The permanent complications rates of thyroidectomies vary greatly in the literature. Permanent postoperative recurrent nerve injury occurs in approximately 0.2%–6.6% of patients and rate of permanent hypoparathyroidism ranges from 0% to 3%.^9^ An experienced and careful specialist will inflict fewer complications.^10^ How is it possible to claim that there are no differences in complications rates, between the new approaches and the standard approach, in events that are not so common and the rates are between such a large range? In our opinion, the verification of this statement would be very difficult and would require a prospective and randomized study with such a large number of patients, that to our knowledge has not yet been done.

Also, a high-volume specialist did not finish his/her training in recent years, has thousands of open thyroidectomies performed, and would have to learn these new techniques with an expected rise in complications in the learning period, putting patients at unnecessary and avoidable risks. Would the patients be willing, knowing that there is a 95% chance that the scar would be invisible or have a very good appearance in a not so long period of time?

Thompson^11^: 128–129 stated “In 30 years of practice, I have not seen major concern for a well-placed, carefully executed, Kocher collar incision/scar. Patients generally are more concerned about getting rid of their cancer, no matter how indolent and curable it may be”; the authors agree with him.

## Financial support

None.

## Declaration of competing interest

The authors declare no conflicts of interest.
